# Hierarchical representation for PPI sites prediction

**DOI:** 10.1186/s12859-022-04624-y

**Published:** 2022-03-20

**Authors:** Michela Quadrini, Sebastian Daberdaku, Carlo Ferrari

**Affiliations:** 1 Department of Information Engineering, University of Padua, Via Gradenigo 6/A, 35131 Padua, Italy; 2 Sorint.Tek, Sorint.LAB group, Via Giovanni Savelli 102, 35129 Padua, Italy; 3 School of Science and Tecnology, University of Camerino, Via Madonna delle Carceri, 8, 62032 Camerino, Italy

**Keywords:** Protein–protein interaction, Hierarchical representation, Graph convolutional networks

## Abstract

**Background:**

Protein–protein interactions have pivotal roles in life processes, and aberrant interactions are associated with various disorders. Interaction site identification is key for understanding disease mechanisms and design new drugs. Effective and efficient computational methods for the PPI prediction are of great value due to the overall cost of experimental methods. Promising results have been obtained using machine learning methods and deep learning techniques, but their effectiveness depends on protein representation and feature selection.

**Results:**

We define a new abstraction of the protein structure, called *hierarchical representations*, considering and quantifying *spatial and sequential neighboring* among amino acids. We also investigate the effect of molecular abstractions using the Graph Convolutional Networks technique to classify amino acids as interface and no-interface ones. Our study takes into account three abstractions, *hierarchical representations, contact map, and the residue sequence*, and considers the eight functional classes of proteins extracted from the Protein–Protein Docking Benchmark 5.0. The performance of our method, evaluated using standard metrics, is compared to the ones obtained with some state-of-the-art protein interface predictors. The analysis of the performance values shows that our method outperforms the considered competitors when the considered molecules are structurally similar.

**Conclusions:**

The *hierarchical representation* can capture the structural properties that promote the interactions and can be used to represent proteins with unknown structures by codifying only their sequential neighboring. Analyzing the results, we conclude that classes should be arranged according to their architectures rather than functions.

**Supplementary Information:**

The online version contains supplementary material available at 10.1186/s12859-022-04624-y.

## Background

Proteins are macromolecules made of one or more sequences of amino acids that fold back on themselves by determining three-dimensional conformations, or shapes, to enable their biological function. Proteins perform a broad range of tasks within organisms, including structural support, signal transmission, immune defense, transport, storage, biochemical reaction catalysis, motility processes. Most of these activities are carried out by interacting with other molecules, including other proteins, RNAs or DNAs, and small ligands [1]. The interactions between two proteins, known as *protein–protein interactions (PPIs)*, determine the metabolic and signaling pathways [2], whose dysfunction or malfunction, as well as alterations in protein interactions, cause several diseases, with the most notable ones being neurodegenerative disorders [3] and cancer [4].

The fast, correct, and reliable identification of PPI sites facilitates understanding the role a protein has in the different biological functions and helps the understanding of the molecular mechanisms of diseases with direct applications in the discovery of new drugs [5–7]. Since experimental methods, including NMR and X-ray crystallography, are labor-intensive, time-consuming, and have high costs, computational methods to predict the PPI sites play a fundamental role. These methods can be roughly divided into sequence-based, structure-based, and hybrid. The sequence-based ones employ information derived from the amino acid sequence alone and use various physico-chemical properties of residues to identify the interface regions. Examples of these features are interface propensity, hydrophobicity, and electrostatic desolvation. However, structural attributes, such as secondary structure and solvent accessibility, are predicted from sequences. A detailed description of the most significant sequence-based methods is reported in [8]. On the other hand, structural-based approaches extract information from the protein shape. The features include solvent-accessible surface area, secondary structure, crystallographic B-factors, and local geometries. Finally, the hybrid methods combine both sequence and structure-derived information.

Several types of computational approaches have been proposed for the PPIs prediction. Among the sequence-based methods, the representative ones include PPiPP [9], PSIVER [10], DLPred [11], NPS-HomPP [12], and LSTM-PHV [13]. PPiPP predicts PPIs by using the position-specific scoring matrix (PSSM) and amino acid composition; PSIVER takes advantage of the PSSM and predicted accessibility as input for a Naive Bayes classifier. DLPred uses a long-short term memory (LSTM) neural network to learn features such as PSSM, physical properties, and hydropathy index. NPS-HomPPI infers interfacial residues from the ones of homologous interacting proteins. LSTM-PHV uses the long short-term memory model with the word2vec embedding and represents the amino acid sequence context as words.

However, it is apparent that more information is required to achieve higher accuracy in predicting PPIs: structural features are important discriminative attributes. In [14], You et al. proposed an approach that transforms the PPI network into a low-dimensional metric space and predicts the PPI sites based on the similarity between the points in the embedded space. In [15], Guo et al. defined a method based on autocovariance coding and support vector machine algorithm. Zhang et al. proposed PredUs, an interactive web server for interfaces prediction based on structural neighbors and a measure of structural similarity of protein structure [16]. With the same aim, Kufareva et al. developed a method based on local statistical properties of the protein surface derived at the level of atomic groups [17]. PrISE uses only the interface structure for template identification, which increases its prediction coverage [18]. On the other hand, some methods based on protein structures take advantage of sequence features. Daberdaku and Ferrari proposed a method based on molecular surface representations that use Zernike descriptors enriched with a chosen subset of physico-chemical properties [19, 20]. Finally, SPPIDER [21] uses the relative solvent accessibility to sequence together with structural features.

Most of the described approaches employ classic machine learning algorithms, including support vector machines, neural networks, and k-Nearest Neighbor. Recent developments of neural networks include deep learning techniques, which have been successfully applied for PPI prediction. Representative sequence-based methods take advantage of Recursive Neural Network architecture [22], a stacked autoencoder [23], and Multimodal Deep Polynomial Network [24]. Structure-based methods are usually based on graphs, such as Convolutional Neural Networks [25, 26] and Graph Convolutional Networks [27, 28]. Although structure-based and hybrid methods are generally more accurate than sequence-based ones, their applicability is limited because they require knowledge of protein structures. Instead, most of the proteins, especially those involved in transient binding interactions or the engineering phase, do not have experimentally determined 3D configurations. In the PPI prediction, one of the biggest challenges for the graph-based deep learning methods is to abstract the proteins to capture the conformational aspects that change when proteins interact with their binding partners [29]. Structural features extracted from unbound proteins may not exist in bound complexes due to conformational changes induced by or required in the binding. In the literature, the performances of methods trained on the bound versions of proteins usually are better than ones obtained by considering unbound proteins [20].

This work mainly focuses on the challenge of proteins representations in the case of partner-independent predictions of interfaces, i.e., the prediction is carried out on the single proteins without any knowledge of the potential binding partner. We consider known experimentally-determined three-dimensional structures, in both unbound and bound versions. To face the protein representations challenge, we abstract the protein shapes into a low-dimensional metric space, referred to as hierarchical representation. This representation formalizes the hierarchical nature of proteins. The protein consists of a amino acids sequence (also called primary structure) that folds back into local and functional patterns (or secondary structure). Such local motifs arrange themselves into global configurations (domains and tertiary structures) to enable their biological tasks. This description shows an intrinsic hierarchy because the secondary structure contains all amino acids precedence, i.e., the primary structure, equipped with local spatial knowledge. Moreover, the tertiary structure formalizes all information related to the sequence of amino acids and the local spatiality. To formalize such a hierarchy that characterizes the protein shapes, we introduce the *sequential* and *spatial neighborhood* relationships among amino acids.

Two residues are sequential neighbors if they are consecutive in the sequence, or spatial neighbors if their two alpha carbons ($C_{\alpha }$) are located at distance smaller than a given threshold.

To predict the interaction sides, we designed a *hybrid method*, called HSS-PPI. It exploits the Graph Convolutional Networks, a deep learning framework [30], as a computational approach. We used eight physico-chemical features to represent the molecular physico-chemical aspects. We also consider the more common structural features according to the biological hypothesis related to the link between shape and function. The physico-chemical indexes are selected from the AAindex1 dataset, a database of numerical value [31]. By using a consensus fuzzy clustering method on all available indices in the AAindex1, Saha et al. identified three high quality subsets (HQIs) of all available in-dices, namely HQI8, HQI24 and HQI40 [32]. The features of the HQI8 amino acid index set were employed in this work. In [20], these features were shown to adequately discriminate interface patches from non-interface ones in bound and unbound Ab structures.

The structural features are the solvent-accessible surface area, relative solvent accessible surface area, Torsion angles PHI and PSI, and the number of residue contacts. Thanks to this molecular abstraction, the proposed approach can be trained on molecules with known structures, as well as on ones with unknown or partially known three-dimensional structures. This way, distinctively from the other methods in the literature [33], the proposed computation approach enables us to use the structural knowledge from other molecules to predict the PPIs of molecules with unknown spatial configurations.

Our study, based on the idea introduced in [34], takes into account the eight classes of proteins extracted from the Protein–Protein Docking Benchmark 5.0 [35], and considers four reasonable values (6Å, 8Å, 10Å, and 12Å) for the distance threshold to abstract the proteins. To investigate the effect of different representations, we applied the framework using hierarchical protein representations, contact mapping, and, finally, the residue sequence. The prediction results were evaluated using six metrics, namely: the area under the receiver operating characteristic curve (AU-ROC), the accuracy, the precision, the recall, the F-measure, and the Matthews correlation coefficient. Finally, such results are compared to the ones obtained with some state-of-the-art protein interface predictors (SPPIDER, PrISE, and NPS-HomPPI).

## Materials and methods

The prediction of PPI sites is a classification procedure of nodes in a graph: each node represents a single amino acid of a protein. The aim is to assign a label, either 1 (interface) or 0 (no-interface), to each node. To address the problem, we take advantage of GCNs. We consider dimers from the Protein–Protein Docking Benchmark 5.0 (DB5) to make a reasonable comparison with the existing PPIs prediction results.

### Benchmark dataset

The data used for the analysis are the Protein–Protein Docking Benchmark 5.0 (DB5). It consists of 230 complexes for bound and unbound versions. Each complex is made up of at least 30 amino acids, characterized by a resolution greater than 3.25 Å. To build our datasets, we divided these complexes according to the eight functional classes proposed by the DB5. The classes are (1) Antibody-Antigen (A), (2) Antigen-Bound Antibody (AB), (3) Enzyme-Inhibitor (EI), (4) Enzyme Substrate (ES), (5) Enzyme complex with a regulatory or accessory chain, (ER) (6) Others, G-protein containing (OG), (7) Others, Receptor containing (OR), (8) and Others, miscellaneous (OX). For each class, we separated the bound version from the unbound one. Moreover, we split the receptors from ligands. In this way, for each functional group, we consider four datasets: unbound ligands, bound ligands, unbound receptors, and bound receptors. We prepared these datasets to understand the most suitable representation for different protein complex classes. To make a reasonable comparison with the existing PPIs prediction approaches, we split the data into training and test sets following [20]. Furthermore, we randomly split the training set into two parts (training set and validation set) as shown in the Additional file 1. In these datasets, we consider amino acids as interface residues if they had at least one heavy (non-hydrogen) atom within 5Å from any heavy ones of the other protein (the same threshold used in [20]).

### Hierarchical representations

Protein representation is one of the challenges in graph-based deep learning method applications. The challenge consists of an abstraction of the protein shape, usually formalized in terms of atomic coordinates and represented as PDBx/mmCIF, PDB, or XML files. In the literature, a common abstraction codifies the distance between every pair of residues determined using Euclidean distance using a binary matrix, the so-called *contact map* [36]. Let *i* and *j* be two residues of a given protein, the respective element $m_{i,j}$ of the matrix is equal to 1 if the two residues are closer than a predetermined threshold, and 0 otherwise. Different contact definitions have been proposed, including the distance between the alpha carbon ($C_{\alpha }$) atoms with threshold 6–12 Å, the distance between the beta carbon ($C_{\beta }$) atoms with cut-offs ranging from 6 to 12Å ($C_{\alpha }$ is used for Glycine), and the distance between the side-chain centers of mass.

Considering the hierarchy of protein shapes, we introduce two relationships, spatial and sequential neighboring, among amino acid pairs. Each residue pair is sequential neighboring if the two amino acids are consecutive in the sequence; otherwise, the two amino acids are spatial neighboring if their Euclidean distance is less than a fixed threshold. The two relationships allow us to distinguish chemical bonds of the primary sequence from the other ones established during the folding process and depended on the amino acid distance. Taking into account this observation, we define the hierarchical representation by quantifying the spatial and sequential neighboring relations starting from the protein PDB file. Let *i* and *j* be two residues of a given protein, we assign 1 if they are sequential residues in the chain, $1/(1+x)$ if *x* if the Euclidean distance between the respective $C_{\alpha }$ atoms of the resides is less than a predetermined threshold, or 0 otherwise. The definition can be summarized as follows

1 $$\begin{aligned} a_{i, j} \! = \! {\left\{ \begin{array}{ll} 1 &{} \textit{if i and j are sequential neighboring residues,}\\ \frac{1}{1+x} &{} \textit{if i and j are spatial neighboring residues,} \\ 0 &{} \textit{otherwise.} \end{array}\right. } \end{aligned}$$

As a consequence, each value $a_{i, j}$ is between 0 and 1. By exploiting the order of amino acids imposed by the primary structure, we can uniquely arrange such values into a matrix: the adjacency matrix of a weighted undirected graph with the nodes being the residues, and the weighted edges representing the relationships among them. This approach allows us to formalize proteins whose 3D structure is known together with molecules with unknown spatial configuration by codifying only the sequential neighboring. Such an aspect is crucial, for example, when a protein is engineered, and the entire structure is still unknown.

#### Input features

The features play a fundamental role in deep learning-based classification procedures. As mentioned in the Background, PPI hybrid methods use structural and physico-chemical features. We extract biochemical properties from AAindex, a database of physico-chemical and biochemical indices of amino acids and amino acid pairs published in the literature [31]. Recently, these indices have been used in different bioinformatics tasks related to proteins, including linear B-cell epitome identification [37] and proinflammatory peptide [38]. The AAindex1 section of this database consists of 566 indices. Three subsets of AAindex1 indices, namely HQI8, HQI24, and HQI40, are generated by considering the centers of 8, 24, and 40 clusters, respectively, computed using a consensus procedure over a fuzzy clustering method as in Saha et al. [32]. In this work, we consider the HQI8, reported in Table 1.

**Table 1:** HQI8 indices

Entry name	Description
BLAM930101	Alpha helix propensity of position 44 in T4 lysozyme
BIOV880101	Information value for accessibility; average fraction 35%
MAXF760101	Normalized frequency of alpha-helix
TSAJ990101	Volumes including the crystallographic waters using the ProtOr
NAKH920108	AA composition of MEM of multi-spanning proteins
CEDJ970104	Composition of amino acids in intracellular protein (percent)
LIFS790101	Conformational preference for all beta-strands
MIYS990104	Optimized relative partition energies - method C

Together with the such physico-chemical and biochemical information, we extract structural features for each protein. In particular, we consider

- *Accessible surface area* (ASA), the protein surface area accessible to the surrounding solvent;
- *Relative accessible surface area* (rASA), a degree of residue solvent exposure [39];
- *Contact number*, the number of spatial neighboring of residues;
- *Torsion angles* $\Phi$ *and* $\Psi$: the description of the rotations of the polypeptide backbone around the bonds between *N*-$C_{\alpha }$ and $C_{\alpha }$-*C*, respectively;
- *Amino acid types*, the amino acid’s identity or type.

These numerical indices form the feature vectors of our model based on the graph convolutional networks architecture. The first eight components of the vectors are the physico-chemical and biochemical indices reported in Table 1, followed by four real numbers corresponding to structural features (ASA, rASA, Contact number, and Torsion angles). Finally, the last twenty values are the components of the one-hot encoding vectors representing the types of amino acids in the protein. For these reasons, the first eight and the last twenty values of the vectors are the same for a particular type of amino acid. Instead, the remaining elements are related to the structure and are different for each residue in the sequence

### Graph convolutional networks

Graph Convolutional Network is a neural network architecture proposed by Kipf and Welling [30]. The architecture works on graphs and takes advantage of their structural information aggregating them on each node from its neighborhoods in a convolutional fashion. Let ${\mathcal {G}}(V, E, w)$ be an undirected weighted graph, where $V = \{v_1, v_2, \dots v_n\}$ is the set of *n* nodes, $E \subset V\times V$ is the set of *m* edges, and $w: E \longrightarrow [0, 1]$ is a weight function that for each pair of *E* associates a number (weight) of the interval [0, 1]. Let *A* be the symmetric matrix, the so-called adjacency matrix, uniquely associated with the graph ${\mathcal {G}}$, whose element $a_{i,j} \in [0,1]$. For each graph ${\mathcal {G}}$, we associate a matrix $X \in {\mathbb {R}}^{n\times m}$, whose *m* rows represent the feature values to associate with the corresponding nodes. Finally, let $L \in \lbrace 0, 1\rbrace ^{n}$ be the vector of labels.

The GCN model aims to learn a function to predict the labels on each node. The model takes the adjacency matrix *A* of graph ${\mathcal {G}}$ and the input feature matrix *X* as input. Each layer of the architecture is defined in terms of the following propagation rule (a non-linear function)

2 $$\begin{aligned} Z^{(h+1)} = f(Z^{(h)}, A), \ \ h = 1, \dots , H, \end{aligned}$$

where $Z^{(0)} = X$ is the input feature matrix. Each layer $Z^{(h)}$ corresponds to a feature matrix, whose rows correspond to the features representing the corresponding nodes. Each layer aggregates these features to form the next layer’s features using the propagation rule *f*. The propagation rule used in this framework is

3 $$\begin{aligned} f(Z^{(h)}, A) = \sigma \left( {\hat{D}}^{-\frac{1}{2}} (A+I) {\hat{D}}^{-\frac{1}{2}} Z^{(h)} W^{(h)} \right) , \end{aligned}$$

where *I* is the identity matrix, and ${\hat{D}}$ is the node degree of $A+I$. ${\hat{D}}$ is a diagonal matrix, whose elements $d_{i,i}$ equal the number of incident edges of node $v_i$ incremented by one. $W^{(h)}$ is the weight matrix for layer *h*, and $\sigma$ is a non-linear activation function. In this work, the Rectified Linear Unit (ReLU) function is appliyed. The feature aggregation for each node is calculated using the following vector equation

4 $$\begin{aligned} g^{(h+1)}_{v_i} = \sigma \left( \sum _{j} \frac{1}{c_{ij}} g^{(l)}_{v_j} W^{(l)}\right) \end{aligned}$$

where *j* iterates over the neighboring nodes of $v_i$, and $c_{ij}$ is a normalization constant obtained from the adjacency matrix to account for the degree difference between $v_i$ and $v_j$.

### HSS-PPI, a hybrid method based on protein shape and sequence for PPI site prediction

**Fig. 1:**
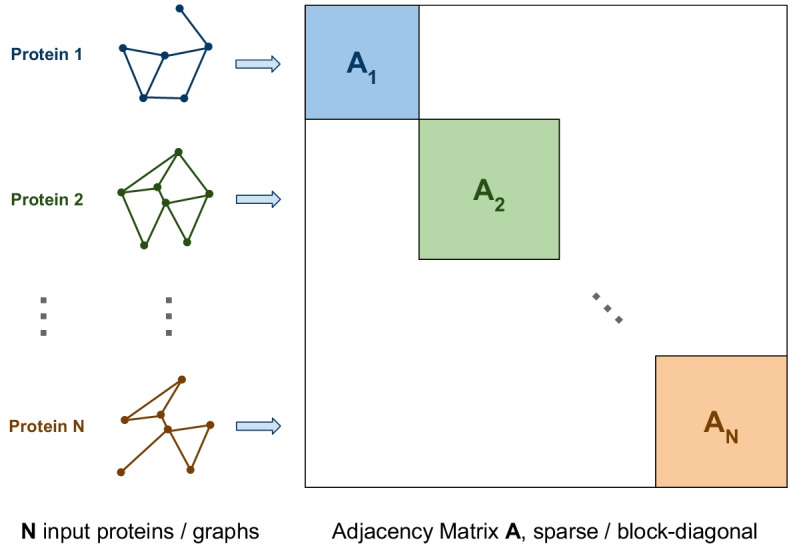
Adjacency graph construction for a given set of proteins

We designed HSS-PPI, a hybrid method based on protein shape and sequence for PPI site prediction. Our overall framework consists of three steps. The first one is to abstract the protein formalized in terms of atomic coordinates of the PDB file. The proposed graph-based abstractions are the hierarchical structure, the contact map, and the sequence: each one of them can be represented by an adjacency matrix. As illustrated on the left of Fig. 1, we associate the adjacency matrix of the selected abstraction to each protein.

**Fig. 2:**
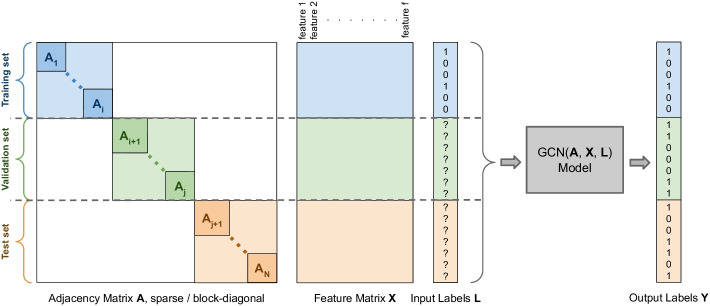
PPI interface residue classification with a semi-supervised GCN framework

Such matrices are the blocks of the sparse block-diagonal adjacency matrix, represented on the right of Fig. 1, which is the input of our model, as shown on the left of Fig. 2. The composite adjacency and feature matrices are split into training, validation, and testing sets using the corresponding row indices as boundaries. The second step consists in adding structural features to the representation. Each protein feature is formalized as a vector. The feature vectors are concatenated to obtain the respective feature matrices, as shown in the center of Fig. 2. In the third and final step, we use the graph convolutional network technique to predict the site of PPIs, as represented on the left of Fig. 2. The model classifies the labels of the nodes in the validation and test sets in a semi-supervised fashion, since only the labels of the elements belonging to the training set are provided as an input to the GCN model.

We compare the performance of the approach with other state-of-the art methods, one proposed by Daberdaku and Ferrari [20], NPS-HomPPI [12], PrISE [18], and SPIDER [21, 40]. As detailed discussed in the Results section, the performance values of these competitors are taken from [20].

The method proposed by Daberdaku and Ferrari [20] takes into account the molecular surface representations for describing protein structure. It considers local surface descriptors based on 3D Zernike moments to identify potential binding sites. These descriptors, which are invariant to roto-translations, are extracted from the protein surface and are enriched with eight physico-chemical properties. Furthermore, it uses Support Vector Machines as a classifier to distinguish interacting local surfaces from non-interacting ones. NPS-HomPPI [12] is a homology-based method that can be used to predict interface residues without any knowledge of the interaction partner. It is based on similarity criteria required for accurate homology-based inference of interface residues in query protein sequence homologs based on the assumption that homologs share significant similarities in sequence, structure, and functional sites. Moreover, NPS-HomPPI classifies the templates into either Safe, Twilight, or Dark Zone, and uses multiple templates from the best available zone to infer interfaces for query proteins. PrISE [18] is a local structural surface similarity-based computational method for predicting the PPI sites. This method represents each local surface structure by using “structural elements”. Each of them consists of a central residue and its surrounding surface neighbors that are represented by their atomic composition and accessible surface areas. The approach decomposes molecular surfaces into many structural elements and searches these elements into pre-calculated databases for similar structural elements with experimentally determined interface information. Finally, it weighs them according to their similarity with the structural elements of the query protein. SPPIDER [21, 40] identifies and recognizes the interface residues site by integrating enhanced relative solvent accessibility (RSA) predictions with high resolution structural data. The approach is based on the concept of “fingerprint” that is derived from the difference between the predicted and actual relative accessible surface area (rASA) of residues as features for interface prediction. Furthermore, SPPIDER uses a consensus method that combines the output of 10 Neural Networks with the majority voting to merge the most informative features into the final predictor.

### Implementation

The framework is implemented in Python using Biopython and TensorFlow 2.0 [41, 42]. We use the PDBParser and DSSP modules of BioPython to abstract the shape into a graph and extract the structural features, respectively. The URL https://gitlab.com/sebastiandaberdaku/hss-ppi points to the dataset and source code that form our framework. The framework was tested on an HPC Server with eight 12-Core Intel Xeon Gold 5118 CPUs running at 2.30 GHz and using 1.5 TB RAM. We set 32 parallel threads under OS Fedora Linux 25. We used Stochastic Gradient Descent as an optimization algorithm, with a learning rate and a dropout value equal to 0.001 and 0.5, respectively. Moreover, we set two hidden layers with 35 and 32 features. Empirical observation during the experimental face helped us in setting 1500 as the maximum number of reached epochs: the training can stop earlier if the performance on the validation set stopped improving. Moreover, we train our deep learning framework on three protein abstractions, the contact map, the residue sequence, and the hierarchical representation, considering distance thresholds of 6 Å, 8 Å, 10 Å, and 12 Å.

### Performance evaluation

Predicting interfacial residues can be formulated as a binary classification problem where each protein residue can be either interfacial or non-interfacial. We evaluate the performance of our approach and compare it with one of some other methods in the literature using six evaluation metrics: *Accuracy* (Acc), *Precision* (P), *Recall* (R), *F-measure* ($\hbox {F}_1$), the *area under the receiver operator characteristic curve (AUC)*, *Matthews correlation coefficient (MCC)*

$$\begin{aligned} \text {Acc}= & {} \frac{\text {TP}+\text {TN}}{\text {TP}+\text {TN}+\text {FP}+\text {FN}}\\ \text {P}= & {} \frac{\text {TP}}{\text {TP}+\text {FP}}\\ \text {R}= & {} \frac{\text {TP}}{\text {TP}+\text {FN}}\\ \text {F}_1= & {} 2 \times \frac{\text {P} \times \text {R}}{\text {P} + \text {R}}\\ \text {MCC}= & {} \frac{\text {TP}\times \text {TN}\! - \! \text {FP}\times \text {FN}}{\sqrt{(\text {TP} \! + \! \text {FP})(\text {TP} \! + \! \text {FN})(\text {TN} \! + \! \text {FP})(\text {TN} \! + \! \text {FN})}} \end{aligned}$$

where *TP* represents the number of interaction sites identified correctly (true positive), *FN* denotes the number of interaction sites identified incorrectly (false negative), *FP* represents the number of non-interaction sites identified incorrectly (false positive), *TN* denotes the number of non-interaction sites identified correctly (true negative). As mentioned earlier, the prediction interface is an imbalanced learning problem. Therefore, F-measure, MCC, and AUC are the three most important evaluation metrics as they can provide morecomprehensive measures than other evaluation metrics [43].

## Results

### Comparison with other methods and discussion

The performance results, evaluated using six metrics (F1 score, Accuracy, Precision, Recall, MCC, AU-ROC) for our method, HSS-PPI, are presented in Tables 3 and 4. We compare these results with the performance obtained by competitors, one proposed by Daberdaku and Ferrari [20], NPS-HomPPI [12], PrISE [18], and SPIDER [21, 40]. Since our organization of the molecules in testing, training and validation sets is coherent to that proposed in the literature, we can take the performance values of the competitors directly from [20], where the performance of the proposed approaches is quantified only in terms of AUC-ROC. This aspect is not a limitation to comparing the methods since the prediction of PPI interface sites is a highly imbalanced classification problem. The AUC-ROC is a comprehensive comparison metric independent of any decision boundaries. Moreover, it is robust to class imbalance. Dataset sizes are shown in Table 2.

**Table 2:** Number of complexes and in each class of the Dataset

Class	Data partition	Complex	Positive		Negative	
			b (%)	u (%)	b (%)	u (%)
$A_r$	Train	8	8	9	92	91
	Validation	3	9	10	91	90
	Test	7	8	10	92	90
$A_l$	Train	9	14	16	86	84
	Validation	3	14	15	86	85
	Test	8	14	15	86	85
$AB_r$	Train	4	9	11	91	89
	Validation	3	8	9	92	91
	Test	5	8	9	92	91
$AB_l$	Train	4	13	13	87	87
	Validation	3	14	13	86	87
	Test	4	16	16	84	84
$EI_r$	Train	18	15	15	85	85
	Validation	12	15	16	85	84
	Test	14	15	16	85	84
$EI_l$	Train	16	29	33	71	67
	Validation	12	32	34	68	66
	Test	16	30	32	70	68
$ER_r$	Train	14	13	13	87	87
	Validation	3	12	12	88	88
	Test	9	11	11	89	89
$ER_l$	Train	10	20	21	80	79
	Validation	5	26	22	74	78
	Test	11	25	22	75	78
$ES_r$	Train	7	9	12	91	88
	Validation	3	10	12	90	88
	Test	7	11	12	89	88
$ES_l$	Train	7	25	21	75	79
	Validation	3	25	23	75	77
	Test	6	22	21	78	79
$OG_r$	Train	8	9	9	91	91
	Validation	3	10	9	90	91
	Test	8	12	12	88	88
$OG_l$	Train	9	24	24	76	76
	Validation	2	22	21	78	79
	Test	7	19	20	81	80
$OR_r$	Train	10	14	13	86	87
	Validation	4	12	11	88	89
	Test	9	13	14	87	86
$OR_l$	Train	9	23	23	77	77
	Validation	5	21	21	79	79
	Test	10	23	24	77	76
$OX_r$	Train	17	16	15	84	85
	Validation	11	15	15	85	85
	Test	19	14	13	86	87
$OX_l$	Train	16	18	18	82	82
	Validation	14	19	20	81	80
	Test	20	20	21	80	81

Positive examples are residue pairs that participate in the interface, negative examples are pairs that do not

HSS-PPI performs better than the competitor predictors in the bound and unbound versions of classes $A_l$, $AB_l$, and $OG_l$. It is an expected result since the ligands are small molecules that adapt their shape to interact with the receptor partner, even though they preserve their native architecture determined by the folding process. Therefore, methods based on the internal conformation of three-dimensional structures are more suitable than the approach based on shapes or homology like PrISE and NPSHomPPI, respectively. However, in the bound version of class $A_l$, our method achieves a ROC-AUC of $65.1\%$, while, for the competitors, the maximum ROC-AUC is $63.0\%$ obtained by SPPIDER. Similarly, for class $A_l$, our method achieves a ROC-AUC of $71.3\%$, while, for the competitors, the maximum ROC- AUC is $62.6\%$ for NPS-HomPPI. The ROC-AUC values obtained with our method take into account the contact map abstraction of proteins and considers the threshold equals 6Å and 8Å for bound and unbound versions, respectively. However, our method achieves higher ROC-AUC values than those obtained with competitive predictors independently of the representation chosen and the threshold value. The AUC-ROC values obtained with the different abstractions and thresholds are comparable, except for the contact map representation with the cut-off equals to 12Å. Also, the values of other metrics (F1 and MCC) are comparable for the bound and unbound versions of this class. However, the results obtained with the hierarchical representation are more reliable than ones obtained for contact maps. Noticeably better prediction performance is achieved in the unbound and the bound versions of class $AB_l$. In fact, for the bound version, the ROC-AUC of our method is equal to $82.6\%$, and the maximum ROC-AUC among considered competitors is $68.3\%$ (PrISE). For the unbound version, the ROC-AUC obtained with our predictor is equal to $78.7\%$, and the maximum ROC-AUC value achieved with the considered competitors is $71.3\%$ (NPS-HomPPI). The ROC-AUCs obtained with our methods take advantage of contact map representation to abstract the molecules, and the threshold equals 12Å. However, as for the $A_l$ class, our method outperforms the competitors regardless of representation and threshold value. In the bound and unbound versions of protein class $OG_l$, our method achieves a ROC-AUC of $77.4\%$ and $69.1\%$, respectively. The maximum achieved ROC-AUC values from the competitors is $72.2\%$ for the unbound (Daberdaku et al.) and $72.2\%$ for the unbound version (NPS-HomPPI). For this class, the values related to HSS-PPI are obtained by abstracting the proteins using the hierarchical representation with the threshold set to 8Å. In the bound version of class $OG_l$, HSS-PPI achieves a ROC-AUC of $75.8\%$, while, for the competitors, the maximum ROC-AUC is $70.7\%$, obtained by the method proposed by HPS-HomPPI. In the unbound version of the protein class $OR_l$, our method achieves a ROC-AUC of $68.4\%$, while, for the competitors, the maximum ROC-AUC is $72.2\%$ obtained with NPS-HomPPI. Similar to class $A_l$, the AUC-ROC values are comparable, except for the contact map using 12Å as the threshold value. The F1 and MCC values related to hierarchical representation are comparable for the bound and unbound versions of this class, although the values related to hierarchical representation are better than ones obtained for contact maps. Our prediction method is comparable for $EI_l$, and $OR_l$. In the bound and unbound versions of class $EI_l$, our method achieves ROC-AUC values equal to $71.5\%$ and $62.6\%$, respectively, while the maximum ROC-AUC values are $75.5\%$ and $74.4\%$ obtained by SPPIDER and NPS-HomPPI, respectively. For the unbound version of this class, ROC-AUC values obtained with hierarchical representations outperform the ones related to the contact maps. In the bound and unbound versions of class $OR_l$, the best ROC-AUC values of our method are equal to $70.3\%$ and $68.3\%$, both obtained with the hierarchical representation. Among the competitors, the best AUC-ROC value for the bound version is $72.3\%$ obtained by the method proposed by Daberdaku and Ferrari, while the best AUC-ROC value for the unbound version is $68.2\%$ obtained by NPS-HomPPI. For this class, the F1 and MCC values obtained with our method using hierarchical representation outperform the ones obtained by considering the contact maps regardless of the chosen threshold. Our prediction method underperformed compared to the competitors in the bound and unbound versions of the classes $ER_l$, $ES_l$, and $OX_l$.

The molecules of these classes show several different architectures, as reported in Tables 4 and 5 of the Additional file 1. Therefore, the internal conformations of three-dimensional structures differ from each other.

The homology-based methods are better performing than the approaches based on shapes or internal conformation. As shown in Table 3, the structural information of the molecules does not improve the performance of the methods. Moreover, we observe that the values of AUC obtained using the sequence are comparable to ones obtained by representing the proteins as contact maps and hierarchical. This aspect could depend on the datasets, formed by molecules with the same biological function rather than structures.

**Table 3:** Measures of F1 score, classification accuracy, precision, recall, MCC and ROC-AUC obtained on the test set of the ligands classes

	A class—ligands											
	F1		Accuracy		Precision		Recall		MCC		AUC-ROC	
	b	u	b	u	b	u	b	u	b	u	b	u
*HSS-PPI*												
Residue Sequence	0.208	0.271	0.731	0.480	0.180	0.170	0.261	0.749	0.054	0.118	0.588	0.613
Contact Map 6 Å	0.266	0.259	0.297	0.302	0.158	0.155	0.919	0.943	0.074	0.108	**0.651**	0.704
Contact Map 8 Å	0.254	0.238	0.201	0.180	0.149	0.140	0.981	0.979	0.027	0.019	0.649	0.713
Contact Map 10 Å	0.252	0.240	0.199	0.224	0.148	0.141	0.966	0.939	0.028	0.030	0.625	0.697
Contact Map 12 Å	0.259	0.252	0.299	0.289	0.154	0.151	0.848	0.883	0.036	0.060	0.589	0.664
Hierarchical Representation 6Å	0.282	0.264	0.445	0.344	0.174	0.159	0.806	0.918	0.127	0.117	0.635	0.661
Hierarchical Representation 8Å	0.272	0.243	0.340	0.192	0.163	0.143	0.896	0.975	0.099	0.028	0.633	0.670
Hierarchical Representation 10Å	0.272	0.243	0.380	0.204	0.164	0.143	0.839	0.978	0.091	0.038	0.624	0.685
Hierarchical Representation 12Å	0.267	0.237	0.340	0.161	0.160	0.139	0.850	0.983	0.060	0.003	0.611	0.684
*Other methods*												
Daberdaku *et. al.*	0.093	0.097	0.811	0.059	0.067	0.052	0.182	0.987	0.019	-0.016	0.538	0.473
SPPIDER											0.630	0.575
NPS-HomPPI											0.610	0.626
PrISE											0.622	0.569

	AB class—ligands											
	F1		Accuracy		Precision		Recall		MCC		AUC-ROC	
	b	u	b	u	b	u	b	u	b	u	b	u
*HSS-PPI*												
Residue Sequence	0.295	u	0.668	u	0.270	u	0.359	u	0.092	u	0.593	u
Contact Map 6 Å	0.325	0.465	0.415	0.715	0.247	0.372	0.792	0.625	0.132	0.292	0.733	0.743
Contact Map 8 Å	0.421	0.496	0.767	0.669	0.333	0.373	0.584	0.746	0.311	0.316	0.760	0.782
Contact Map 10 Å	0.403	0.482	0.715	0.571	0.311	0.431	0.635	0.713	0.315	0.217	0.826	0.777
Contact Map 12 Å	0.416	0.519	0.663	0.550	0.317	0.388	0.673	0.911	0.247	0.286	0.826	0.786
Hierarchical Representation 6Å	0.348	0.447	0.294	0.689	0.221	0.352	0.975	0.651	0.105	0.269	0.723	0.698
Hierarchical Representation 8Å	0.278	0.493	0.395	0.700	0.194	0.375	0.762	0.725	0.083	0.329	0.728	0.737
Hierarchical Representation 10Å	0.263	0.286	0.410	0.800	0.169	0.501	0.750	0.213	0.066	0.218	0.761	0.743
Hierarchical Representation 12Å	0.262	0.274	0.411	0.789	0.167	0.383	0.750	0.214	0.080	0.175	0.769	0.752
*Other methods*												
Daberdaku et. al.	0.183	0.115	0.653	0.246	0.112	0.063	0.553	0.931	0.110	0.071	0.655	0.667
SPPIDER											0.573	0.556
NPS-HomPPI											0.676	0.713
PrISE											0.683	0.649

	EI class—ligands											
	F1		Accuracy		Precision		Recall		MCC		AUC-ROC	
	b	u	b	u	b	u	b	u	b	u	b	u
*HSS-PPI*												
Residue Sequence	0.414	0.399	0.415	0.482	0.287	0.292	0.792	0.678	0.038	0.053	0.601	0.565
Contact Map 6 Å	0.405	0.406	0.511	0.557	0.317	0.349	0.730	0.675	0.124	0.121	0.715	0.644
Contact Map 8 Å	0.343	0.387	0.545	0.483	0.242	0.281	0.637	0.752	0.080	0.059	0.687	0.636
Contact Map 10 Å	0.429	0.399	0.343	0.403	0.285	0.274	0.931	0.830	0.025	0.020	0.616	0.575
Contact Map 12 Å	0.422	0.393	0.308	0.397	0.278	0.268	0.947	0.830	-0.001	0.016	0.594	0.592
Hierarchical Representation 6Å	0.420	0.417	0.608	0.422	0.337	0.287	0.634	0.826	0.174	0.027	0.688	0.592
Hierarchical Representation 8Å	0.393	0.394	0.621	0.540	0.310	0.305	0.597	0.682	0.168	0.092	0.696	0.616
Hierarchical Representation 10Å	0.364	0.375	0.625	0.595	0.284	0.337	0.557	0.612	0.137	0.127	0.694	0.626
Hierarchical Representation 12Å	0.343	0.358	0.501	0.588	0.233	0.318	0.683	0.585	0.044	0.109	0.686	0.624
*Other methods*												
Daberdaku *et. al.*	0.253	0.203	0.535	0.360	0.154	0.118	0.793	0.865	0.167	0.086	0.725	0.673
SPPIDER											0.755	0.732
NPS-HomPPI											0.701	0.744
PrISE											0.719	0.678

	ER class—ligands											
	F1		Accuracy		Precision		Recall		MCC		AUC-ROC	
	b	u	b	u	b	u	b	u	b	u	b	u
*HSS-PPI*												
Residue Sequence	0.386	0.331	0.356	0.339	0.260	0.230	0.879	0.853	0.076	0.071	0.593	0.597
Contact Map 6 Å	0.364	0.281	0.357	0.392	0.243	0.230	0.829	0.639	0.033	- 0.018	0.529	0.518
Contact Map 8 Å	0.370	0.281	0.321	0.392	0.244	0.230	0.886	0.639	0.012	-0.018	0.510	0.518
Contact Map 10 Å	0.382	0.327	0.270	0.233	0.251	0.224	0.976	0.928	0.036	0.012	0.506	0.523
Contact Map 12 Å	0.334	0.283	0.335	0.242	0.257	0.219	0.841	0.856	0.036	0.005	0.530	0.558
Hierarchical Representation 6Å	0.391	0.295	0.468	0.482	0.278	0.272	0.762	0.635	0.085	0.078	0.585	0.577
Hierarchical Representation 8Å	0.369	0.303	0.436	0.399	0.254	0.266	0.769	0.724	0.075	0.079	0.556	0.560
Hierarchical Representation 10Å	0.347	0.268	0.452	0.430	0.240	0.255	0.724	0.589	0.054	0.005	0.532	0.526
Hierarchical Representation 12Å	0.345	0.237	0.438	0.410	0.242	0.238	0.730	0.544	0.030	-0.043	0.524	0.485
*Other methods*												
Daberdaku *et. al.*	0.214	0.137	0.494	0.087	0.136	0.077	0.851	0.998	0.167	0.019	0.774	0.685
SPPIDER											0.778	0.740
NPS-HomPPI											0.643	0.761
PrISE											0.700	0.680

	ES class—ligands											
	F1		Accuracy		Precision		Recall		MCC		AUC-ROC	
	b	u	b	u	b	u	b	u	b	u	b	u
*HSS-PPI*												
Residue Sequence	0.301	0.309	0.533	0.379	0.214	0.199	0.568	0.797	0.069	0.059	0.578	0.552
Contact Map 6 Å	0.333	0.330	0.487	0.359	0.232	0.217	0.708	0.862	0.127	0.089	0.620	0.601
Contact Map 8 Å	0.318	0.315	0.222	0.219	0.198	0.196	0.995	0.995	0.061	0.059	0.611	0.607
Contact Map 10 Å	0.317	0.322	0.227	0.247	0.196	0.201	0.995	0.995	0.068	0.098	0.616	0.620
Contact Map 12 Å	0.302	0.331	0.189	0.272	0.182	0.207	1.000	0.994	0.025	0.107	0.620	0.639
Hierarchical Representation 6Å	0.320	0.323	0.523	0.534	0.227	0.237	0.633	0.615	0.102	0.114	0.602	0.591
Hierarchical Representation 8Å	0.305	0.305	0.482	0.482	0.221	0.221	0.636	0.636	0.093	0.093	0.590	0.590
Hierarchical Representation 10Å	0.323	0.310	0.359	0.270	0.222	0.200	0.824	0.914	0.103	0.040	0.597	0.573
Hierarchical Representation 12Å	0.314	0.323	0.279	0.233	0.202	0.204	0.914	0.995	0.079	0.074	0.617	0.587
*Other methods*												
Daberdaku *et. al.*	0.150	0.169	0.665	0.636	0.087	0.102	0.665	0.670	0.142	0.148	0.703	0.720
SPPIDER											0.778	0.740
NPS-HomPPI											0.643	0.671
PrISE											0.700	0.680

	OG class—ligands											
	F1		Accuracy		Precision		Recall		MCC		AUC-ROC	
*HSS-PPI*												
Sequence Å	0.285	0.249	0.641	0.699	0.206	0.232	0.486	0.278	0.114	0.055	0.605	0.552
Contact Map 6 Å	0.329	0.367	0.398	0.472	0.203	0.242	0.933	0.871	0.181	0.178	0.722	0.659
Contact Map 8 Å	0.317	0.329	0.352	0.540	0.196	0.261	0.935	0.730	0.140	0.150	0.703	0.684
Contact Map 10 Å	0.304	0.193	0.313	0.741	0.191	0.245	0.923	0.230	0.103	0.110	0.712	0.677
Contact Map 12 Å	0.211	0.299	0.669	0.555	0.184	0.203	0.420	0.656	0.106	0.092	0.659	0.677
Hierarchical Representation 6Å	0.360	0.397	0.567	0.551	0.241	0.270	0.786	0.837	0.230	0.229	0.749	0.684
Hierarchical Representation 8Å	0.352	0.381	0.455	0.491	0.224	0.258	0.918	0.871	0.227	0.194	0.758	0.683
Hierarchical Representation 10Å	0.335	0.072	0.403	0.775	0.215	0.086	0.922	0.065	0.185	0.001	0.744	0.678
Hierarchical Representation 12Å	0.312	0.207	0.345	0.750	0.194	0.280	0.929	0.226	0.137	0.098	0.721	0.684
*Other methods*												
Daberdaku *et. al.*	0.127	0.108	0.373	0.089	0.069	0.058	0.927	0.996	0.125	0.037	0.622	0.653
SPPIDER											0.663	0.659
NPS-HomPPI											0.707	0.722
PrISE											0.693	0.658

	OR class—ligands											
	F1		Accuracy		Precision		Recall		MCC		AUC-ROC	
*HSS-PPI*												
Residue Sequence	0.398	0.387	0.410	0.547	0.282	0.335	0.873	0.611	0.128	0.107	0.598	0.591
Contact Map 6 Å	0.191	0.263	0.582	0.709	0.138	0.374	0.407	0.276	-0.002	0.175	0.577	0.656
Contact Map 8 Å	0.238	0.220	0.653	0.630	0.356	0.355	0.343	0.249	0.112	0.052	0.646	0.648
Contact Map 10 Å	0.221	0.435	0.627	0.349	0.220	0.317	0.360	0.986	0.053	0.045	0.671	0.594
Contact Map 12 Å	0.220	0.436	0.504	0.350	0.244	0.313	0.502	1.000	0.035	0.043	0.665	0.568
Hierarchical Representation 6Å	0.338	0.393	0.725	0.583	0.392	0.378	0.359	0.602	0.194	0.186	0.656	0.660
Hierarchical Representation 8Å	0.343	0.376	0.672	0.574	0.388	0.398	0.437	0.576	0.194	0.199	0.607	0.681
Hierarchical Representation 10Å	0.263	0.35	0.732	0.522	0.455	0.391	0.284	0.606	0.201	0.176	0.692	0.682
Hierarchical Representation 12Å	0.269	0.239	0.712	0.704	0.342	0.402	0.375	0.254	0.207	0.180	0.704	0.676
*Other methods*												
Daberdaku *et. al.*	0.172	0.190	0.269	0.592	0.098	0.153	0.980	0.593	0.120	0.095	0.723	0.658
SPPIDER											0.698	0.613
NPS-HomPPI											0.681	0.691
PrISE											0.698	0.601

	OX class—ligands											
	F1		Accuracy		Precision		Recall		MCC		AUC-ROC	
*HSS-PPI*												
Residue Sequence	0.347	0.360	0.310	0.242	0.218	0.230	0.935	0.986	0.059	0.007	0.565	0.536
Contact Map 6 Å	0.218	0.347	0.268	0.265	0.128	0.222	0.926	0.950	0.064	0.006	0.632	0.552
Contact Map 8 Å	0.335	0.356	0.207	0.249	0.207	0.228	1.000	0.968	0.000	-0.002	0.553	0.537
Contact Map 10 Å	0.335	0.361	0.207	0.246	0.207	0.230	1.000	0.981	0.000	0.004	0.568	0.543
Contact Map 12 Å	0.335	0.358	0.207	0.251	0.207	0.229	1.000	0.969	0.000	0.001	0.578	0.553
Hierarchical Representation 6Å	0.344	0.348	0.455	0.265	0.231	0.222	0.743	0.950	0.080	0.006	0.588	0.569
Hierarchical Representation 8Å	0.348	0.348	0.384	0.266	0.226	0.222	0.846	0.950	0.073	0.008	0.578	0.556
Hierarchical Representation 10Å	0.340	0.346	0.372	0.262	0.222	0.221	0.833	0.950	0.046	-0.002	0.567	0.551
Hierarchical Representation 12Å	0.332	0.346	0.379	0.262	0.226	0.221	0.804	0.950	0.043	-0.002	0.564	0.542
*Other methods*												
Daberdaku *et. al.*	0.168	0.151	0.369	0.352	0.095	0.085	0.896	0.846	0.120	0.077	0.720	0.668
SPPIDER											0.726	0.688
NPS-HomPPI											0.646	0.655
PrISE											0.712	0.662

The proposed methodology performs better than the competitor predictors for bound and unbound classes $A_r$, $AB_r$, $EI_r$, and $OG_r$. Like to the $A_l$, $AB_l$, and $OG_l$ classes, this result was expected since the molecules of $A_r$, $AB_r$, classes shown the same architecture, while the architectures of the test set for $EI_r$, and $OG_r$ classes are well represented in the training and validation sets. As a consequence, our method is more appropriate than the competitors. In the bound and unbound version of class $A_r$, our method achieves a ROC-AUC of $97.6\%$ and $98.0\%$, respectively. The maximum achieved ROC-AUC values from the competitors are $95.4\%$ for the unbound and $93.9\%$ for the bound version. Both values are obtained by the method proposed by Daberdaku and Ferrari. The ROC-AUC values obtained with HSS-PPI (our method) take into account the contact map abstraction of proteins with the threshold equals 8Å. Our method achieves ROC-AUCs that are better than ones obtained with the competitor predictors regardless of the choice of representation and threshold value. On the other hand, the value of F1, MCC, and AUC-ROC obtained with hierarchical representation mainly outperform the ones related to the abstractions based on the contact map. In the bound version of class $AB_r$, our method achieves a ROC-AUC of $95.8\%$, while, for the competitors, the maximum ROC-AUC is $89.0\%$ obtained by the method proposed by Daberdaku and Ferrari. Similarly, in the unbound version of class $AB_r$, our method achieves a ROC-AUC of $96.5\%$, while, for the competitors, the maximum ROC- AUC is $84.5\%$ for Daberdaku and Ferrari’s method. The ROC-AUC values obtained with our method take into account the contact map abstraction of proteins and consider the threshold equals 10Å. The values of other metrics (F1 and MCC) related to hierarchical representation are mainly better than ones obtained for contact maps. In the bound and unbound version of class $EI_r$, our method achieves a ROC-AUC of $77.0\%$ and $74.5\%$, respectively. The maximum ROC-AUC values of competitors are $76.4\%$ and $74.7\%$. These values are obtained by the methods proposed by Daberdaku and Ferrari. The ROC-AUC values related to our method take into account hierarchical representation as protein abstraction and considers the threshold equals 10Å, while the F1 and MCC values are comparable. In the bound version of class $OG_r$, our method achieves a ROC-AUC of $75.6\%$, while, for the competitors, the maximum ROC-AUC is $74.8\%$ obtained by the method proposed by SPPIDER. Our method achieves a ROC-AUC of $74.8\%$ for the unbound version, which is comparable with the ROC-AUC of $76.8\%$ obtained by NPS-HomPPI. However, for both bound and unbound versions, the F1 and MCC values obtained by using the hierarchical representation outperform the value related to the contact map. The results of our prediction method are comparable with the competitors for proteins of class $ER_r$ in the bound and unbound versions, while our prediction method underperformed compared to the competitors in the bound and unbound versions of the classes $ES_r$, $OG_r$, and $OX_r$. Observing Table 4, we note that the values of AUC obtained using the sequence are comparable or better than ones obtained by representing the proteins as contact maps and hierarchical. Hence, as observed for ligands, we could suppose that also the elements in $ES_r$, $OG_r$, and $OX_r$ exhibit more sequential similarity than the structural one.

**Fig. 3:**
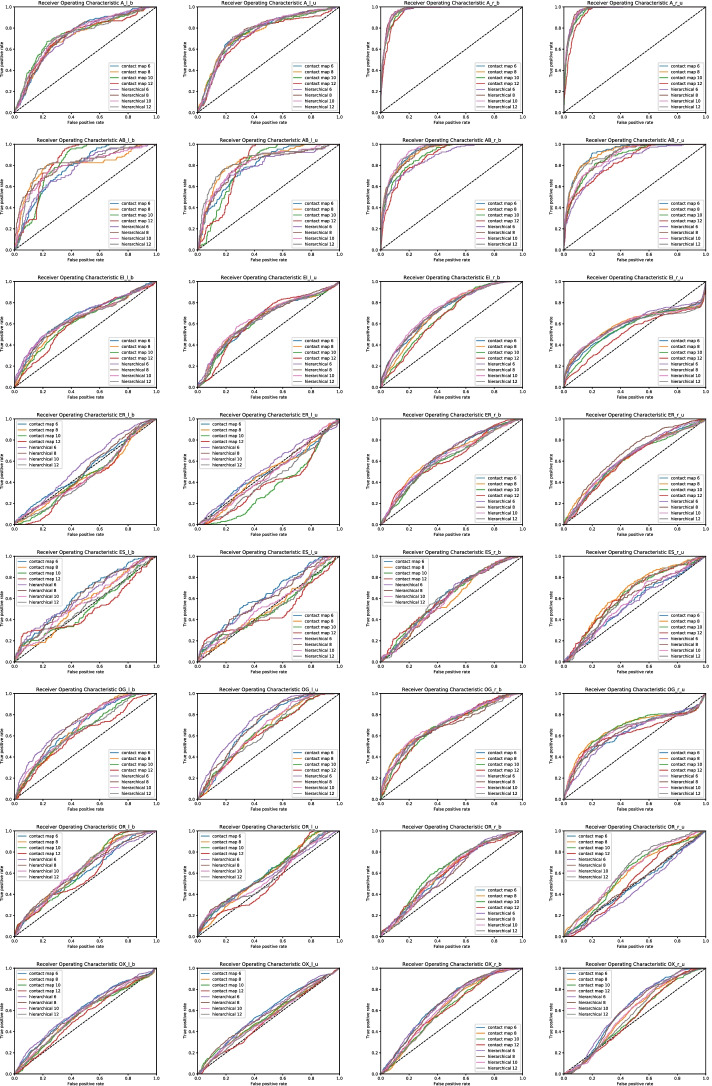
Average Receiver Operating Characteristic curve comparison of the proposed PPI interface prediction method by using hierarchical representation, contact map and sequence as protein representation with different thresholds (6Å, 8Å, 10Å, 12Å) for each protein class

**Fig. 4:**
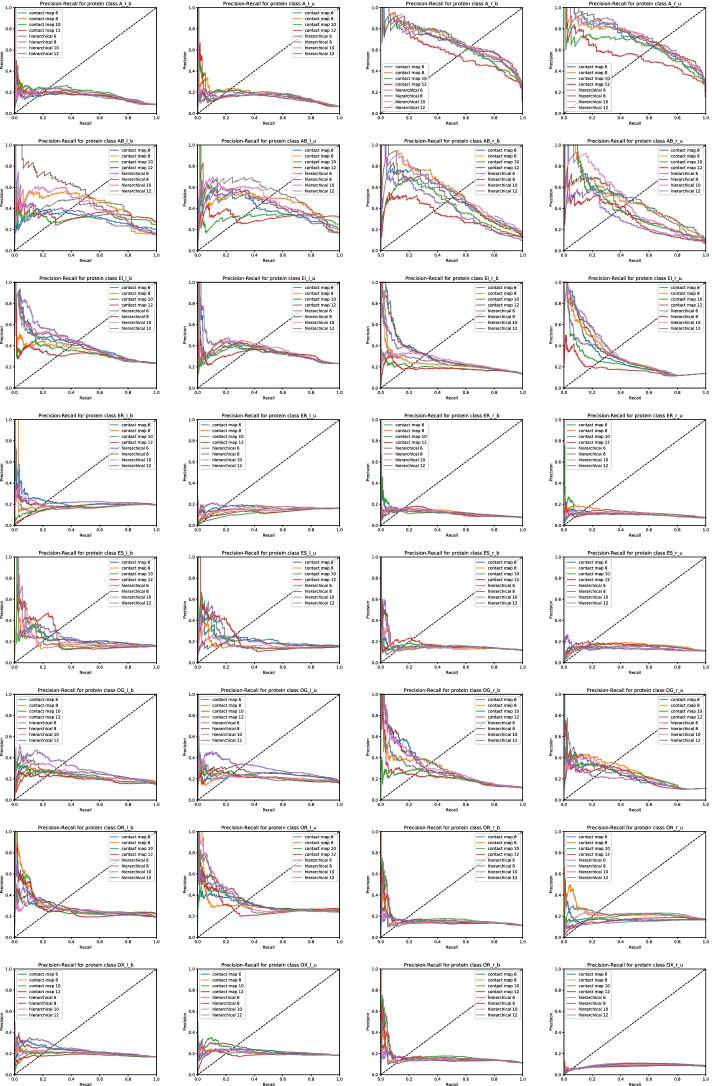
Average Precision-Recall curve comparison of the proposed PPI interface prediction method by using hierarchical representation, contact map and sequence as protein representation with different thresholds (6Å, 8Å, 10Å, 12Å) for each protein class

**Table 4:** Measures of F1 score, classification accuracy, precision, recall, MCC and ROC-AUC obtained on the test set of the receptors classes

	A class—receptors											
	F1		Accuracy		Precision		Recall		MCC		AUC-ROC	
*HSS-PPI*												
Residue Sequence	0.328	0.345	0.851	0.857	0.243	0.265	0.534	0.548	0.287	0.306	0.845	0.835
Contact Map 6 Å	0.622	0.629	0.947	0.930	0.631	0.526	0.665	0.864	0.608	0.631	0.975	0.980
Contact Map 8 Å	0.630	0.620	0.950	0.925	0.658	0.502	0.660	0.889	0.620	0.624	0.974	0.978
Contact Map 10 Å	0.578	0.628	0.920	0.940	0.467	0.602	0.823	0.724	0.576	0.617	0.970	0.975
Contact Map 12 Å	0.552	0.580	0.932	0.920	0.523	0.483	0.645	0.801	0.535	0.574	0.966	0.967
Hierarchical Representation 6Å	0.657	0.592	0.952	0.910	0.667	0.447	0.701	0.951	0.647	0.609	0.971	0.978
Hierarchical Representation 8Å	0.647	0.665	0.950	0.945	0.663	0.608	0.690	0.803	0.637	0.659	0.976	0.980
Hierarchical Representation 10Å	0.619	0.632	0.952	0.927	0.718	0.510	0.595	0.907	0.615	0.638	0.975	0.978
Hierarchical Representation 12Å	0.619	0.604	0.948	0.918	0.656	0.474	0.647	0.910	0.611	0.612	0.975	0.977
*Other methods*												
Daberdaku *et. al.*	0.272	0.274	0.862	0.876	0.166	0.169	0.917	0.883	0.346	0.341	0.954	0.939
SPPIDER											0.773	0.754
NPS-HomPPI											0.796	0.780
PrISE											0.770	0.758

	AB class—receptors											
	F1		Accuracy		Precision		Recall		MCC		AUC-ROC	
*HSS-PPI*												
Residue Sequence	0.212	0.244	0.886	0.750	0.265	0.167	0.214	0.537	0.168	0.188	0.727	0.737
Contact Map 6 Å	0.432	0.400	0.930	0.930	0.535	0.391	0.425	0.443	0.422	0.382	0.942	0.939
Contact Map 8 Å	0.300	0.316	0.929	0.931	0.496	0.466	0.276	0.297	0.323	0.327	0.945	0.955
Contact Map 10 Å	0.406	0.293	0.924	0.926	0.589	0.304	0.391	0.302	0.424	0.291	0.958	0.965
Contact Map 12 Å	0.357	0.359	0.909	0.914	0.488	0.317	0.455	0.454	0.370	0.353	0.956	0.964
Hierarchical Representation 6Å	0.428	0.347	0.921	0.913	0.501	0.383	0.446	0.423	0.411	0.332	0.886	0.886
Hierarchical Representation 8Å	0.450	0.407	0.921	0.925	0.491	0.566	0.492	0.484	0.431	0.417	0.918	0.928
Hierarchical Representation 10Å	0.412	0.415	0.930	0.931	0.601	0.615	0.424	0.473	0.417	0.429	0.948	0.953
Hierarchical Representation 12Å	0.281	0.340	0.923	0.929	0.439	0.469	0.264	0.341	0.292	0.348	0.955	0.963
*Other methods*												
Daberdaku *et. al.*	0.230	0.228	0.910	0.913	0.161	0.156	0.590	0.546	0.256	0.250	0.890	0.845
SPPIDER											0.757	0.783
NPS-HomPPI											0.701	0.698
PrISE											0.776	0.789

	EI class—receptors											
	F1		Accuracy		Precision		Recall		MCC		AUC-ROC	
*HSS-PPI*												
Residue Sequence	0.281	0.292	0.590	0.646	0.190	0.224	0.596	0.505	0.125	0.133	0.631	0.622
Contact Map 6 Å	0.303	0.349	0.827	0.713	0.344	0.275	0.326	0.610	0.231	0.262	0.753	0.732
Contact Map 8 Å	0.364	0.363	0.725	0.722	0.283	0.280	0.604	0.594	0.270	0.271	0.753	0.729
Contact Map 10 Å	0.327	0.378	0.734	0.720	0.299	0.310	0.521	0.580	0.233	0.281	0.755	0.708
Contact Map 12 Å	0.270	0.330	0.733	0.605	0.235	0.232	0.440	0.643	0.167	0.189	0.732	0.678
Hierarchical Representation 6Å	0.329	0.342	0.817	0.744	0.320	0.289	0.369	0.507	0.237	0.236	0.749	0.725
Hierarchical Representation 8Å	0.354	0.364	0.797	0.776	0.331	0.331	0.446	0.511	0.267	0.285	0.766	0.732
Hierarchical Representation 10Å	0.363	0.380	0.806	0.753	0.388	0.338	0.445	0.574	0.290	0.304	0.770	0.745
Hierarchical Representation 12Å	0.414	0.362	0.760	0.668	0.348	0.275	0.611	0.681	0.324	0.278	0.783	0.732
*Other methods*												
Daberdaku *et. al.*	0.156	0.148	0.604	0.645	0.089	0.087	0.770	0.705	0.158	0.146	0.764	0.747
SPPIDER											0.696	0.673
NPS-HomPPI											0.720	0.682
PrISE											0.755	0.700

	ER class—receptors											
	F1		Accuracy		Precision		Recall		MCC		AUC-ROC	
*HSS-PPI*												
Residue Sequence	0.207	0.187	0.588	0.193	0.133	0.107	0.550	0.966	0.076	0.049	0.608	0.572
Contact Map 6 Å	0.186	0.211	0.106	0.691	0.106	0.157	1.000	0.480	0.000	0.115	0.635	0.663
Contact Map 8 Å	0.186	0.158	0.106	0.808	0.106	0.173	1.000	0.239	0.000	0.090	0.638	0.648
Contact Map 10 Å	0.186	0.179	0.106	0.103	0.106	0.102	1.000	1.000	0.000	0.000	0.655	0.634
Contact Map 12 Å	0.186	0.179	0.106	0.101	0.106	0.101	1.000	1.000	0.000	0.000	0.693	0.614
Hierarchical Representation 6Å	0.202	0.184	0.326	0.151	0.117	0.105	0.854	0.988	0.048	0.041	0.626	0.654
Hierarchical Representation 8Å	0.186	0.194	0.106	0.329	0.106	0.114	1.000	0.841	0.000	0.059	0.615	0.671
Hierarchical Representation 10Å	0.186	0.195	0.106	0.245	0.106	0.112	1.000	0.929	0.000	0.039	0.590	0.654
Hierarchical Representation 12Å	0.186	0.173	0.106	0.675	0.106	0.151	1.000	0.408	0.000	0.054	0.596	0.655
*Other methods*												
Daberdaku *et. al.*	0.145	0.109	0.733	0.747	0.089	0.065	0.580	0.465	0.136	0.092	0.734	0.663
SPPIDER											0.731	0.736
NPS-HomPPI											0.588	0.589
PrISE											0.742	0.674

	ES class—receptors											
	F1		Accuracy		Precision		Recall		MCC		AUC-ROC	
*HSS-PPI*												
Residue Sequence	0.257	0.232	0.512	0.278	0.163	0.137	0.663	0.879	0.097	0.070	0.596	0.565
Contact Map 6 Å	0.228	0.247	0.693	0.617	0.180	0.167	0.351	0.542	0.070	0.111	0.608	0.608
Contact Map 8 Å	0.241	0.302	0.586	0.623	0.176	0.210	0.534	0.627	0.097	0.186	0.612	0.645
Contact Map 10 Å	0.255	0.292	0.621	0.601	0.215	0.201	0.524	0.672	0.131	0.192	0.621	0.660
Contact Map 12 Å	0.263	0.239	0.642	0.326	0.211	0.143	0.511	0.829	0.140	0.064	0.626	0.658
Hierarchical Representation 6Å	0.204	0.248	0.748	0.478	0.187	0.155	0.248	0.689	0.064	0.102	0.607	0.580
Hierarchical Representation 8Å	0.236	0.261	0.678	0.663	0.185	0.184	0.388	0.511	0.080	0.135	0.621	0.604
Hierarchical Representation 10Å	0.245	0.284	0.673	0.545	0.192	0.181	0.416	0.752	0.094	0.185	0.620	0.631
Hierarchical Representation 12Å	0.230	0.272	0.717	0.436	0.229	0.167	0.353	0.825	0.114	0.154	0.625	0.651
*Other methods*												
Daberdaku *et. al.*	0.031	0.121	0.954	0.861	0.086	0.090	0.023	0.281	0.023	0.096	0.712	0.709
SPPIDER											0.742	0.727
NPS-HomPPI											0.604	0.654
PrISE											0.742	0.664

	OG class—receptors											
	F1		Accuracy		Precision		Recall		MCC		AUC-ROC	
*HSS-PPI*												
Residue Sequence	0.276	0.248	0.721	0.603	0.222	0.178	0.384	0.491	0.122	0.072	0.596	0.572
Contact Map 6 Å	0.389	0.288	0.755	0.806	0.298	0.295	0.617	0.313	0.285	0.196	0.756	0.711
Contact Map 8 Å	0.305	0.274	0.580	0.532	0.210	0.176	0.705	0.735	0.163	0.157	0.728	0.730
Contact Map 10 Å	0.300	0.259	0.685	0.603	0.252	0.199	0.444	0.580	0.128	0.129	0.682	0.735
Contact Map 12 Å	0.266	0.190	0.431	0.602	0.168	0.126	0.735	0.515	0.059	0.083	0.688	0.704
Hierarchical Representation 6Å	0.359	0.244	0.744	0.772	0.281	0.230	0.550	0.324	0.240	0.140	0.717	0.662
Hierarchical Representation 8Å	0.355	0.292	0.706	0.794	0.253	0.274	0.647	0.386	0.241	0.209	0.743	0.707
Hierarchical Representation 10Å	0.351	0.351	0.709	0.752	0.257	0.266	0.602	0.605	0.221	0.278	0.764	0.748
Hierarchical Representation 12Å	0.323	0.326	0.692	0.778	0.239	0.285	0.555	0.496	0.175	0.259	0.744	0.742
*Other methods*												
Daberdaku *et. al.*	0.184	0.144	0.704	0.805	0.113	0.103	0.552	0.340	0.145	0.100	0.700	0.631
SPPIDER											0.748	0.753
NPS-HomPPI											0.699	0.768
PrISE											0.686	0.673

	OR class—receptors											
	F1		Accuracy		Precision		Recall		MCC		AUC-ROC	
*HSS-PPI*												
Residue Sequence	0.249	0.255	0.229	0.467	0.149	0.187	0.949	0.672	0.040	0.033	0.572	0.547
Contact Map 6 Å	0.191	0.201	0.582	0.540	0.138	0.179	0.407	0.424	-0.002	-0.009	0.577	0.507
Contact Map 8 Å	0.173	0.284	0.683	0.318	0.173	0.193	0.272	0.887	0.021	0.021	0.586	0.494
Contact Map 10 Å	0.207	0.287	0.604	0.315	0.162	0.195	0.440	0.891	0.042	0.017	0.600	0.502
Contact Map 12 Å	0.245	0.288	0.531	0.316	0.167	0.196	0.587	0.897	0.067	0.027	0.621	0.492
Hierarchical Representation 6Å	0.159	0.178	0.676	0.601	0.127	0.162	0.266	0.343	-0.009	-0.012	0.546	0.508
Hierarchical Representation 8Å	0.192	0.198	0.614	0.508	0.141	0.155	0.382	0.432	0.002	-0.077	0.566	0.499
Hierarchical Representation 10Å	0.172	0.211	0.659	0.569	0.146	0.169	0.303	0.400	0.002	-0.049	0.562	0.505
Representation 12Å	0.213	0.276	0.608	0.281	0.162	0.188	0.447	0.846	0.043	-0.031	0.584	0.492
*Other methods*												
Daberdaku *et. al.*	0.121	0.103	0.662	0.115	0.073	0.057	0.558	0.968	0.093	0.024	0.659	0.626
SPPIDER											0.691	0.667
NPS-HomPPI											0.712	0.642
PrISE											0.655	0.642

	OX class—receptors											
	F1		Accuracy		Precision		Recall		MCC		AUC-ROC	
*HSS-PPI*												
Residue Sequence	0.213	0.208	0.219	0.264	0.12459	0.120	0.939	0.901	0.051	0.055	0.583	0.594
Contact Map 6 Å	0.218	0.220	0.268	0.414	0.128	0.140	0.926	0.759	0.064	0.082	0.632	0.619
Contact Map 8 Å	0.226	0.198	0.345	0.437	0.137	0.169	0.858	0.710	0.091	0.073	0.643	0.630
Contact Map 10 Å	0.227	0.186	0.390	0.420	0.139	0.111	0.814	0.707	0.090	0.045	0.636	0.627
Contact Map 12 Å	0.227	0.181	0.440	0.443	0.141	0.108	0.752	0.654	0.089	0.033	0.622	0.619
Hierarchical Representation 6Å	0.216	0.201	0.249	0.167	0.126	0.115	0.928	0.965	0.056	0.025	0.614	0.601
Hierarchical Representation 8Å	0.218	0.215	0.272	0.328	0.128	0.127	0.921	0.854	0.063	0.066	0.620	0.604
Hierarchical Representation 10Å	0.219	0.209	0.272	0.425	0.128	0.137	0.913	0.706	0.058	0.050	0.612	0.606
Hierarchical Representation 12Å	0.215	0.187	0.280	0.499	0.126	0.164	0.880	0.572	0.043	0.042	0.603	0.605
*Other methods*												
Daberdaku *et. al.*	0.108	0.081	0.677	0.670	0.063	0.045	0.555	0.490	0.089	0.056	0.665	0.614
SPPIDER											0.667	0.640
NPS-HomPPI											0.612	0.635
PrISE											0.641	0.592

By analyzing the results, it emerges that the threshold value used to construct contact maps or hierarchical representations dramatically affects the performances of our method in varying ways for different datasets. We note different effects when the threshold value is increased. In some datasets, increasing the threshold value determines an increment of performance, while, in others, this leads to a decrease. Since the cut-off is a purely geometric value, the abstractions can mainly capture the local structural motifs (i.e., elements of secondary structures) if the cut-off is low (i.e., 6–8 Å). On the other hand, a higher cut-off value also captures the geometrical relations of the global structure (i.e., tertiary structure). This additional information starts playing a fundamental role. Our experiments bring out a link between the cut-off and the molecular architectures.

We note that in the dataset whose molecules exhibit the same architecture (for example the class of AB receptors which consists of paired heavy-light chains (H-L) with the same structure [44]), the performance values of the approach increase with an increasing threshold. The performance decreases with an increasing cut-off, for example in the EI receptor dataset (which consists of enzymes), where the molecules of the dataset exhibit many different architectures. The cause is intrinsic to the hierarchical nature of the shape, which is strictly related to the protein’s biological tasks. The elements of secondary structures play a critical role in several functions like PPI interactions [45]. These elements, and, thus, the relative contact maps and hierarchical representations, are similar regardless of their arrangements in their global 3D configuration. Instead, such structures, i.e., tertiary structures, are comparable only among molecules with the same architecture (such $AB_r$ dataset) [46]. Therefore, the threshold increment in the dataset with molecules characterized by heterogeneous architectures results in adding noise, while such increment represents further details in the other case.

Figures 3 and 4 show the Receiver Operating Characteristic curve and Precision Recall curve obtained by the GCN model on each dataset of ligands and receptors, respectively. We have trained the model by using the contact map and hierarchical abstraction with 500 epochs. For each abstraction, we have considered four different thresholds (6Å, 8Å, 10Å, and 12Å).

To interpret the results, we consider the architectures of the ligands and receptors according to the CATH classification. In particular, the architectures of the molecules are Sandwich, 3-Layer(aba) Sandwich, 2 Solenoid, Orthogonal Bundle, Alpha-Beta Complex, Up-down Bundle, Roll, Alpha Horseshoe. The occurrences of these architectures inside the protein classes are not evenly distributed. Tables 4 and 5 reported in the Additional file 1 show the occurrence ratios for these classes. Taking into account such ratios and the performance of our method, we observe a relation between them. For example, we observe that our method achieves optimal results for classes $A_r$ and $AB_r$ , whose molecules mainly show a single architecture, the Sandwich one. Furthermore, our approach does not reach sufficient values for some classes like the $EI_r$, whose molecules show eight different architectures. In particular, the Alpha-Beta Complex and Propeller are two architectures that characterize some molecules in the test set, but they are not present in the training and validation set. Therefore, site prediction of some molecules like 1QQU and 1RGH, classified as easy to predict in the Benchmark, shows low performance. The observation also works for the ligand classes.

To confirm this observation, which may seem like an intuition, we conducted further experimentation. Looking at the Benchmark, we noted the 2-Layer Sandwich architecture spans over all classes of ligands, i.e., 2-Layer Sandwich is the most represented architecture in the Benchmark. Thus, we consider all proteins that exhibit 2-Layer Sandwich architecture despite their biological group. The list of the molecule group is reported in Table 3 in the Additional file 1, while the performance results, evaluated using the six metrics, are presented in Table 5. We observe that the best value of AUC- ROC is 85$\%$, obtained with a threshold equal to 12Å. We also note that this result is the best one among all ligand biological classes. Moreover, it is evident that the threshold value used to construct contact maps or hierarchical representations dramatically changes the performance of our method. We note that in the dataset whose molecules show the same architecture, the performance values of the approach increase with an increasing threshold. Moreover, we observe that the performance decreases with increasing cut-off if molecules of the dataset show many different architectures. These observations lead us to hypothesize that the structural organization of amino acid interactions changes depending on the type of architecture. The increment of the threshold in the dataset with molecules characterized by several architectures may mean adding noises, while such increment represents further information in the other case. These results and observations confirm the biological hypothesis that protein behaviors depend on their three-dimensional conformations. The analysis reveals that organizing the dataset by considering the structural similarity improves the performance of our method. Thus, structural classifications of proteins play a fundamental role, and they can be faced by computation methods without any requirement of biological experiments, which are expensive and time-consuming.

**Table 5:** Measures of F1 score, classification accuracy, precision, recall, MCC and ROC-AUC obtained on the test set of the ligands classes

	F1		Accuracy		Precision		Recall		MCC		AUC-ROC	
	b	u	b	u	b	u	b	u	b	u	b	u
2-layer architecture—ligands												
Contact Map 6 Å	0.390	0.364	0.247	0.232	0.247	0.229	1.00	1.00	0.00	0.014	0.836	0.831
Contact Map 8 Å	0.390	0.356	0.247	0.230	0.247	0.228	1.00	1.00	0.00	0.010	0.768	0.779
Contact Map 10 Å	0.390	0.366	0.247	0.235	0.247	0.229	1.00	1.00	0.00	0.012	0.811	0.765
Contact Map 12 Å	0.390	0.364	0.247	0.228	0.247	0.228	1.00	1.00	0.00	0.00	0.745	0.662
Hierarchical Representation 6Å	0.390	0.385	0.247	0.290	0.247	0.243	1.00	1.00	0.00	0.09	0.790	0.779
Hierarchical Representation 8Å	0.390	0.382	0.247	0.283	0.247	0.241	1.00	1.00	0.00	0.08	0.830	0.815
Hierarchical Representation 10Å	0.390	0.375	0.247	0.263	0.247	0.239	1.00	1.00	0.00	0.06	0.852	0.858
Hierarchical Representation 12Å	0.390	0.364	0.247	0.228	0.247	0.228	1.00	1.00	0.00	0.00	0.871	0.861

These results and observations agree with the biological hypothesis that protein behaviors depend on their three-dimensional conformations. Analyzing the results, it is evident that the performance of our approach can be improved by a structural classification to select the molecules of training and validation sets.

Such structural classification of proteins can be faced by computational methods without requiring any biological experiments which can be expensive and time-consuming.

Another suggestion is related to the features that can be selected from the AAindex databases taking into account the molecular class to capture the physical-chemical characteristics exhibited by the molecules in the diverse interaction mechanisms. Furthermore, the molecular abstraction that we have introduced in terms of spatial and sequential relationships between amino acid is another important feature since it allows us to formalize both proteins whose 3D structure is known both unknown. Such an aspect is crucial, for example, when a protein is engineered, and the entire structure is still unknown since the method can be trained by taking advantages of molecules with known structures.

## Conclusions and future work

In this work, we have focused on the PPI sites prediction by considering their experimentally-determined structures. We have classified the amino acids into interface and no-interface using the Graph Convolutional Networks technique, a deep learning framework. To test the approach, we have applied the framework to the dimers of DB5, divided into eight functional classes. Moreover, we have considered three representations (hierarchical representations, contact maps, and residue sequences). We have also considered different thresholds for the distances between $\hbox {C}_\alpha$ atoms (6Å, 8Å, 10Å, and 12Å). In the literature, other structure-based methods have been proposed and tested [33]. However, the proposed molecular abstraction, obtained by quantifying spatial and sequential relationships among amino acids and referred to as hierarchical structure, is another relevant feature. Thanks to the representation, HSS-PPI trains on molecules with known structures together with ones with unknown or partially known three-dimensional structures. As a result, differentiating from the methods presented in the literature, our approach allows us to consider the structural knowledge of other molecules to predict PPIs of molecules with unknown or partially known spatial configurations. Such an aspect is crucial, for example, when a protein is engineered, and the entire structure is still undetermined. Consequently, we can conclude that the performance depends on the molecules’ structural similarity Therefore, our approach works better on proteins with similar structures rather than similar functions.

As future work, motivated by the results’ analysis, we have planned to apply our framework on paratope interacting residue prediction. Moreover, we have also decided to use our framework considering another classification of the DB5 according to protein three-dimensional structures. In this scenario, motivated by our previous results obtained from RNA secondary structures with pseudoknots comparison [47, 48], we believe that it is important to compare and classify the protein structures considering their tree representations and exploiting edit distance or alignment algorithms. Although some structural classifications have been proposed in the literature, like SCOP [49] or CATH classification [50], our approach, which is an extension of the one proposed to compare RNAs, will work on polynomial time and will neglect the sequence of amino acids.

Motivated by the different performances obtained considering the proposed datasets (corresponding to functional classes of DB5) with a set of fixed features, another important direction is to investigate different feature set by using feature selection methods. In this way, we can consider a set of input variables and choose the more representative quantities for each functional protein group. It is also interesting to select different features for receptors and ligands separately. We will focus our attention on the structural properties by exploring the RNA-based topological methodology introduced in [51]. Finally, another important future direction to explore is to extend the proposed approach to identify the binding partner specificity.

## Supplementary Information

**Additional file 1.** Contains additional information on the used dataset.

## Data Availability

The datasets generated and/or analysed during the current study, the binaries (Linux x64) used to compute the training, validation, and testing samples and the Python scripts are all available at the URL https://gitlab.com/sebastiandaberdaku/hss-ppi.

## Abbreviations

Acc: Accuracy
ASA: Accessible surface area
AU-ROC: Receiver operating characteristic curve
$$C_\alpha$$: Alpha carbons
$$C_{\beta }$$: Beta carbon
DB5: Protein–protein docking benchmark
F1: F-measure
GCN: Graph convolutional network
HQI8: Eight high-quality subsets of indices
MCC: Matthews correlation coefficient
P: Precision
PPIs: Protein–protein interactions
PSSM: Position-specific scoring matrix
rASA: Relative accessible surface area
R: Recall
